# Alcohol consumption, drinking patterns and cancer incidence in an Australian cohort of 226,162 participants aged 45 years and over

**DOI:** 10.1038/s41416-020-01101-2

**Published:** 2020-10-11

**Authors:** Peter Sarich, Karen Canfell, Sam Egger, Emily Banks, Grace Joshy, Paul Grogan, Marianne F. Weber

**Affiliations:** 1 Cancer Research Division, Cancer Council NSW, PO Box 572, Kings Cross, Sydney, NSW 1340 Australia; 2 Sydney School of Public Health, The University of Sydney, Edward Ford Building (A27), Sydney, NSW 2006 Australia; 3 Prince of Wales Clinical School, University of New South Wales, Edmund Blacket Building, Sydney, NSW 2052 Australia; 4 National Centre for Epidemiology and Population Health, Australian National University, Building 62, Canberra, ACT 2601 Australia

**Keywords:** Risk factors, Cancer epidemiology

## Abstract

**Background:**

Although overall alcohol consumption is known to increase the risk of a number of cancers internationally, evidence for Australia and evidence regarding the pattern of drinking and cancer risk is limited.

**Methods:**

Adjusted hazard ratios (HR) and 95% confidence intervals (CI) for cancer risk in relation to overall alcohol consumption (drinks/week) and pattern of drinking were calculated using Cox proportional hazard regressions for 226,162 participants aged ≥45 years (2006–2009) in the 45 and Up Study, an Australian prospective cohort study. Incident primary cancer cases were ascertained by linkage to the New South Wales Cancer Registry to 2013 by the Centre for Health Record Linkage.

**Results:**

Over a median of 5.4 years, 17,332 cancers were diagnosed. Increasing levels of alcohol intake were associated with increased risk of cancers of the upper aerodigestive tract (1.19; 1.10–1.29), mouth and pharynx (1.18; 1.08–1.29), oesophagus (1.22; 1.04–1.43), colorectum (1.09; 1.04–1.15), colon (1.13; 1.06–1.20), liver (1.22; 1.04–1.44) and breast (1.11; 1.02–1.21). Breast cancer risk was marginally associated with drinking pattern, with higher risk when intake was concentrated on 1–3 days/week compared to the same amount spread over 4–7 days (*P*_interaction_ = 0.049).

**Conclusions:**

Alcohol consumption confers a significant risk of cancer, and drinking pattern may be independently related to breast cancer risk.

## Background

Alcohol consumption is commonplace in Australia, with the most recent national survey reporting that 79% of the adult population consumed alcohol in 2017–2018.^1^ Australians aged 15 years and over consumed 10.6 L of alcohol per capita in 2016, which is higher than in the United States and Canada (9.8 L and 8.9 L, respectively), higher than the average for the European Region (9.8 L) and substantially higher than the global average of 6.4 L.^2^ It is also estimated that over a third of Australians aged 15 years and over have a 30-day history of heavy episodic drinking. Alcohol consumption is an important risk factor for cancer and has been estimated to account for 2.8% of cancers in Australia and 5.5% of cancers globally,^3,4^ as well as 4.5% of the cancer burden in Australia.^5^ Studies first identified a relationship between alcohol and cancer as far back as 1903,^6^ and in 1988, the International Agency for Research on Cancer classified alcohol as a Group 1 carcinogen (the highest IARC classification) for cancers of the mouth, pharynx, larynx, oesophagus and liver. In 2010–2012, IARC reviewed the evidence and determined that there is a causal relationship between alcohol and colorectal and female breast cancer.^7,8^ In addition, the World Cancer Research Fund (WCRF) concluded it is probable that consuming >45 g of alcohol per day (i.e., 4.5 standard Australian drinks^9^) increases the risk of stomach cancer, and consuming up to 30 g of alcohol per day decreases the risk of kidney cancer.^10^ Further, both organisations report there is suggestive evidence that heavy drinking causes pancreatic cancer, with recent meta-analyses supporting this conclusion.^11–14^ The evidence for other cancer types is inconclusive, although meta-analyses have reported increased risk of myelodysplastic syndromes,^15^ melanoma^11^ and cancers of the gallbladder,^11^ lung^11^ and prostate^11,16^ with increased intake, and inverse associations with thyroid cancer,^11,17^ Hodgkin lymphoma^11,18^ and non-Hodgkin lymphoma (NHL).^11,19^

The strongest evidence for the link between alcohol and cancer has come predominantly from observational studies that compared drinking and non-drinking cohorts and/or dose–response relationships. Less well studied is the influence of the pattern of drinking.^20,21^ While the types of drinking patterns in the literature have varied greatly,^22,23^ there have been two main patterns of interest in relation to cancer outcomes: (1) frequency of drinking and (2) consumption of a large number of drinks on drinking occasions, which is commonly referred to as ‘heavy episodic’ drinking and/or ‘binge’ drinking.^20,24–38^ A number of cancers have been linked to specific drinking patterns, including IARC Group 1 alcohol-related cancers (mouth, pharynx, oesophagus and breast) and other cancers, including stomach, pancreas, lung and prostate cancer.^24–33^ Large prospective studies that have used a consistent methodology to examine multiple cancer types are few. Previous reports have generally varied with respect to the methodology used to assess the relationship between drinking patterns and a single cancer type, making comparisons between studies difficult.

The relationship between alcohol consumption and cancer risk is likely to vary across different regions of the world,^11^ due in part to regional variation in gene polymorphisms that relate to the metabolism of alcohol.^39^ In Australia, the relationship between alcohol consumption and a number of individual cancer types has been investigated,^40^ but to our knowledge, there are no population-based, prospective studies that quantify the risk of alcohol consumption on many cancer types in a single study, nor are there studies that investigate the impact of drinking pattern on cancer risk. We used a large prospective cohort study in Australia, the 45 and Up Study, to quantify the relationship between self-reported alcohol consumption and primary incident cancer diagnoses captured by record linkage to cancer registry data.

## Methods

### Study sample

The Sax Institute’s 45 and Up Study is a prospective cohort study of 267,153 participants conceived to investigate healthy ageing, with methods previously described.^41^ In summary, men and women aged ≥45 years were randomly sampled from the general population of New South Wales (NSW), Australia, between 2006 and 2009 using the Services Australia (formerly Medicare Australia) enrolment database. The database has records for all Australian citizens and permanent residents, as well as some temporary residents and refugees. Persons aged ≥80 years and persons living in rural and remote areas were oversampled. Participants completed a postal questionnaire (available at https://www.saxinstitute.org.au/our-work/45-up-study/questionnaires/), which contained items on demographic factors, lifestyle and health behaviours (such as smoking, physical activity and cancer screening uptake), and medical history (including the presence of chronic disease and quality of life). Participants completed a 5-year follow-up questionnaire (median 5.3 years after baseline; response rate 53%).

Ethics approval for the 45 and Up Study was provided by the University of NSW Human Research Ethics Committee (reference: 10186) and for this specific analysis by the NSW Population Health Services Research Ethics Committee (reference: 2014/08/551).

### Data linkage

Personal identifiers were used to probabilistically link records from participants in the 45 and Up Study to records from the NSW Cancer Registry (NSWCR) and the NSW Registry of Births Deaths and Marriages by the NSW Ministry of Health’s Centre for Health Record Linkage (CHeReL; http://www.cherel.org.au/). The CHeReL used a best-practice approach in privacy-preserving record linkage^42^ along with the open-source probabilistic record linkage software Choice Maker.^43^ The probabilistic matching process is highly accurate (false-positive and false-negative rates <0.4%) and a detailed explanation of the linkage process has been published elsewhere.^44^

### Cancer and mortality data

Cancer incidence was ascertained from the NSWCR, which captures all primary invasive cancers diagnosed in NSW residents apart from non-melanoma skin cancer. Cancer diagnoses were available from January 1994 to December 2013, and were classified according to the International Classification of Diseases, version 10.^45^ All cancers stated by IARC to be causally related to alcohol consumption were examined separately, along with any cancer type with at least 100 incident cases, including cancers of unknown primary site. Breast cancer was examined for women only. All cancers combined, IARC-determined alcohol-related cancers combined (ARCC; mouth, pharynx, larynx, oesophagus, colorectum, liver and female breast) and non-alcohol-related cancers combined (Non-ARCC; all other cancer types) were also examined. Cancers of the colon and rectum were examined both separately and combined, due to evidence that the risk relationship may differ for these two sites.^8^ Cancers of the mouth and pharynx, oesophagus and larynx were also combined as upper aerodigestive tract cancer. Fact of death was ascertained from the NSW Registry of Births, Deaths and Marriages, to December 2013. Cause of death data was not used for analysis.

### Alcohol consumption

Alcohol consumption was ascertained from two items on the baseline and follow-up questionnaires: ‘About how many alcoholic drinks do you have each week? One drink = a glass of wine, middy of beer or nip of spirits (put ‘0' if you do not drink, or have less than one drink each week)' and, ‘On how many days each week do you usually drink alcohol?'. Participants reported their response as an integer. Responses to these were used to derive two independent variables: (1) to assess the total amount of alcohol consumed in a week, and (2) to assess the pattern of consumption across the week.

The following categories and stratification criteria were defined for each of the independent variables at baseline:

(1) Number of drinks per week (‘weekly alcohol consumption’): ‘0 to <1 drink’, ‘≥1 to ≤3.5 drinks’, ‘>3.5 to ≤7 drinks’, ‘>7 to ≤14 drinks’, ‘>14 to ≤28 drinks’ and ‘>28 drinks’. When analysing breast, endometrial and ovarian cancer ‘>14 drinks/week’ was used as the maximum category as there were few heavy drinkers among women. Light drinkers (≥1 to ≤3.5 drinks per week) were used as the reference group rather than non-drinkers due to the potential for bias from the ‘sick-quitter effect’, whereby non-drinkers may have quit drinking due to symptoms from undiagnosed cancer, which would potentially result in an underestimate of relative risks in relation to heavier drinking.^46,47^ This variable was categorised with cut points aimed to align with the Australian alcohol consumption guidelines to minimise the risk of long-term harm (≤2 standard drinks/day) and short-term harm (≤4 standard drinks/day), where a standard drink contains 10 g of ethanol.^9^

(2) Pattern of drinking:

- ‘≥4 to <7 drinks/week over 4–7 days’ (i.e., participants had between 1 and no more than 1.75 drinks on any one day of the week; reference category)
- ‘≥7 to <14 drinks/week over 4–7 days’ (i.e., ≥1 to <3.5 drinks on any one day of the week)
- ‘≥14 drinks/week over 4–7 days’ (i.e., ≥2 drinks on any one day of the week)
- ‘≥4 to <7 drinks/week over 1–3 days’ (i.e., ≥1.3 to <7 drinks on any one day of the week)
- ‘≥7 to <14 drinks/week over 1–3 days’ (i.e., ≥2.3 to <14 drinks on any one day of the week)
- ‘≥14 drinks/week over 1–3 days’ (i.e., ≥4.7 drinks on any one day of the week)

Participants who consumed <4 drinks/week were excluded from the pattern of drinking analysis. The cut point of 1–3 versus 4–7 days was chosen to classify participants as those who consumed alcohol on more than half or less than half of days of the week, and to capture participants who only drink on the weekend and Friday evening, versus those who also drink during the week.

### Statistical analyses

Participants who had cancer other than non-melanoma skin cancer prior to joining the study (either self-reported or reported in the NSWCR from January 1994) were excluded from analysis. An exception was participants with self-reported melanoma that was not confirmed by the NSWCR, who were included (since skin cancers are known to be self-reported inaccurately).^48,49^ Participants with missing alcohol consumption data or linkage errors were also excluded.

Hazard ratios (HR) and 95% confidence intervals (CI) of a primary cancer diagnosis in relation to weekly alcohol consumption and pattern of drinking were calculated using Cox proportional hazard regressions using age as the underlying time variable.^50^ Censoring occurred at cancer diagnosis, death or the end of study period (December 2013), whichever occurred first. If a participant was diagnosed with more than one primary cancer at different sites, they were counted as a case in the analysis of each cancer type. If a participant reported a hysterectomy, bilateral oophorectomy or radical prostatectomy at baseline, they were excluded from the analysis for endometrium, ovary and prostate cancer, respectively, but were included in the analysis of all cancers combined (regardless of their specific cancer diagnosis).

The risk for each cancer type in relation to weekly alcohol consumption was assessed both categorically, and as a continuous variable in log-linear Cox regressions with hazard ratios representing the change in risk per seven-drink increase in weekly alcohol consumption (among drinkers). For the continuous variable, participants within each category of alcohol consumption at baseline were assigned the mean level of alcohol consumption they reported at the 5-year follow-up questionnaire. This was to allow for regression dilution,^51^ to reduce the potential for misclassification and to mitigate the impact of outliers on the linear trend.

Because sex differences have been reported for alcohol and colorectal cancer risk, and differences between never- and ever-smokers for alcohol and cancers of the mouth, pharynx, oesophagus, liver and larynx,^7,8,11^ two-way statistical interaction tests between alcohol consumption as a continuous variable among drinkers and both sex and smoking status (never-smoking vs. ever-smoking) were conducted for each alcohol-related cancer type and for ARCC, with the inclusion of alcohol consumption as a categorical variable in the model as the main effect. The results were stratified where relevant.

The cumulative absolute risk of cancer diagnosis from age 25 to 85 years in Australia in 2014 by sex and level of alcohol consumption was calculated for each of the IARC-determined alcohol-related cancer types and for these combined (i.e., ARCC). This calculation was based on the results of the continuous variable analysis, and three categories of drinking were used (0 to <1, ≥1 to ≤14 and >14 drinks per week). The methods used to calculate cumulative absolute risk are detailed in the supplementary material.

The risk of all cancers combined, all ARCC and cancers of the colorectum, breast, lung and prostate were assessed in relation to the pattern of drinking. Breast and colorectal cancer were the only alcohol-related cancer types with sufficient cases for analysis, while lung and prostate cancer were included due to prior reports of associations with drinking pattern.^27–29^ A *P* value for interaction by the pattern of drinking was calculated, defined as the test of interaction between days per week and drinks per week.

All regressions were adjusted for potential confounders that were self-reported in the baseline questionnaire, including sex, education, household income, health insurance status, partner status, country of birth, smoking status and intensity, body mass index and physical activity (see Supplementary Table 1). Remoteness was derived from postcode in the Department of Human Services enrolment database, and categories were based on the Accessibility/Remoteness Index for Australia (ARIA+ 2006).^52^ Melanoma risk was additionally adjusted for time spent outdoors and skin tone. Dietary-related variables (fruit, vegetable, fibre, red meat and processed meat consumption) were included as potential confounders for cancer types where the WCRF has found convincing or probable evidence of a causal relationship.^10^ Parity and age at first birth, breastfeeding duration, menopausal status, hormonal contraceptive use and use of menopausal hormone therapy (MHT) were included as potential confounders for cancers of the breast, endometrium and ovary. Hormonal contraceptive use was also included for liver cancer,^53^ and MHT use for colorectal cancer.^54^ Adjustment for aspirin use was made for oesophageal and colorectal cancer.^55^ Adjustment for history of bowel, breast and prostate screening was made for colorectum, breast and prostate cancer, respectively. All potential confounders except sex had a missing indicator category.

A test of the proportional hazard assumption was performed for all Cox regressions. If significant violations were detected, then log–log survival curves stratified by the variables in violation were plotted. If, upon visual inspection, the lines were non-parallel for a covariate, a stratified Cox model was calculated to examine whether the HRs deviated. If the lines were non-parallel for the exposure variable (alcohol consumption), the model was divided into two age groups with equal person-years of follow-up (<60 and ≥60 years) to investigate differences in HRs. If HRs were similar across strata, then models without stratification were reported.

Sensitivity analyses examined the impact of excluding the first year of follow-up (to assess the possibility of reverse causation), and the impact of excluding participants with self-reported melanoma at baseline on estimates of melanoma risk. For the analyses of drinks per week as a continuous variable, sensitivity analyses examined the impact of using the mean number of drinks per week reported at baseline rather than at 5-year follow-up (to assess the impact of regression dilution), and the impact of using the actual number of drinks per week reported by participants rather than the mean of each category of intake.

Analyses were performed using SAS 9.4 and STATA 16.0.

## Results

A total of 226,162 of 267,153 participants in the 45 and Up Study (84.7%) were included for analysis after excluding 268 (0.1%) participants who withdrew their consent to participate after baseline, 175 (0.07%) from a pilot study, 5 (0.002%) aged <45 years at baseline, 36,216 (13.6%) with a previous cancer diagnosis, 4273 (1.6%) with missing information on overall alcohol consumption and 54 (0.02%) with data linkage errors. Of included participants, 17,332 (7.7%) had at least one incident cancer by December 2013 over a median follow-up of 5.4 years.

Overall, 151,685 (67.1%) participants consumed at least one alcoholic drink per week, including 32,647 (14.4%) who consumed >14 drinks per week. The proportion of participants who consumed alcohol at least weekly was higher than in the 2007–2008 Australian National Health Survey^56^ for men aged 45 years and over (e.g., 75.8% vs. 68.2% for men aged 65–74 years), but similar for women (e.g., 53.8% vs. 52.8% for women aged 65–74 years; Supplementary Table 2). Of the 116,389 participants consuming ≥4 drinks per week, 1,177 (1.0%) were excluded from the drinking pattern analyses due to missing information on the number of drinking days per week. Of the 115,212 drinkers included in the drinking pattern analyses, 27,575 (23.9%) consumed alcohol 1–3 days per week.

Among participants who had high numbers of alcoholic drinks per week, a higher proportion were men, Australian-born, consumed processed meat more than once per week and usually spent 2 or more hours outdoors per day, compared to participants who had fewer drinks per week (Table 1). Participants consuming 0 to <1 drink per week tended to be older than drinkers and compared to light drinkers, a higher proportion were current smokers, had inadequate physical activity (according to the Australian physical activity guidelines,^57^) and used aspirin, and a lower proportion had a university degree, a household income ≥ $70,000 per year, private health insurance or were married or living with a partner. Among women who had high numbers of alcoholic drinks per week, a higher proportion were nulliparous, had never breastfed and had ever used hormonal contraceptives compared to women who had fewer drinks per week. A higher proportion of participants who drank on 4–7 days of the week had a history of cancer screening, and a lower proportion were overweight or obese, compared to those who drank on only 1–3 days of the week. Among women who consumed alcohol on more days of the week, a higher proportion were postmenopausal and had ever used MHT compared to those who drank on fewer days of the week.

**Table 1:** Socio-demographic and other characteristics by alcohol consumption in the 45 and Up Study (2006–2013).

	Alcohol consumption (drinks per week)						Drinking days per week^a^	
	0 to <1	≥1 to ≤3.5	>3.5 to ≤7	>7 to ≤14	>14 to ≤28	>28	1–3	4–7
*Characteristics at baseline*								
Participants (*n*)	74,477	35,295	43,769	39,974	23,673	8974	27,575	87,637
Male (%)	32.5	41.3	43.8	53.3	72.2	89.6	56.0	56.5
Mean age in years (SD)	63.4 (11.6)	60.6 (10.6)	61.8 (10.9)	60.9 (10.2)	60.7 (9.8)	60.0 (9.1)	57.7 (9.1)	62.1 (10.5)
Major city resident (%)	53.2	54.3	52.4	51.4	49.0	45.1	53.2	50.1
University degree (%)	17.8	26.6	27.1	28.6	27.1	19.3	24.8	27.8
Household income ≥ $70 000^b^ (%)	14.4	26.8	28.7	33.4	34.9	28.9	34.8	30.7
Private health insurance^c^ (%)	55.4	69.2	72.1	73.2	70.7	59.5	68.5	72.2
Married or living with partner (%)	68.7	76.3	78.7	80.4	80.3	73.6	77.6	79.9
Born in Australia (%)	72.4	72.4	75.4	77.1	78.6	81.6	77.7	77.0
Current smoker (%)	7.7	5.7	5.3	6.9	10.1	18.6	9.3	7.3
Overweight or obese^d^ (%)	56.6	58.0	53.7	56.4	63.0	68.6	63.8	55.7
Inadequate physical activity^e^ (%)	27.3	22.0	18.2	16.1	16.9	21.0	17.9	17.1
<2 fruit serves per day^f^ (%)	37.8	37.3	38.7	44.5	52.9	63.7	42.9	46.4
<5 vegetable serves per day (%)	64.6	66.4	65.4	67.0	70.1	73.3	68.0	67.4
<7 fibre serves per week^g^ (%)	15.1	13.5	11.2	13.5	17.0	27.4	15.5	14.1
Red meat >5 times per week (%)	9.8	8.5	9.1	10.7	14.3	21.5	9.4	12.4
Processed meat >1 time per week (%)	27.3	29.6	30.4	34.2	41.9	51.5	35.6	35.7
≥2 h spent outdoors per day (%)	64.1	67.2	70.5	73.0	77.0	80.7	72.3	73.9
Fair skin tone (%)	69.3	68.6	70.1	70.2	70.7	70.5	68.8	70.9
Nulliparous (women) (%)	9.9	9.7	10.5	12.4	16.4	22.1	11.4	12.4
Never breastfed (women) (%)	24.1	18.9	19.6	21.1	26.2	33.3	21.1	21.2
Postmenopausal (women) (%)	65.5	62.0	64.2	62.3	60.8	53.1	54.0	65.6
Ever-used HC (women) (%)	70.4	82.0	83.5	87.0	89.0	88.7	89.3	84.5
Ever-used MHT (women) (%)	34.4	36.3	38.7	38.6	38.9	36.2	32.2	40.7
Current aspirin use (%)	22.2	19.2	20.1	19.9	21.2	21.2	17.0	21.3
Ever had bowel screening (%)	44.1	46.5	49.5	50.5	51.2	47.1	44.6	51.8
Ever had breast screening (women) (%)	85.4	87.4	89.3	89.0	87.4	82.0	86.3	89.6
Ever had PSA test (men) (%)	65.7	68.0	70.1	70.9	70.1	64.8	66.1	70.9
*Characteristics by the end of follow-up*								
Person-years of follow-up (thousands)^h^	407.3	196.2	242.5	220.4	129.7	49.3	153.0	482.2
Number of incident cancer cases^h^	5421	2417	3212	3241	2166	875	1977	7392

*SD* standard deviation, *HC* hormonal contraceptives, *MHT* menopausal hormone therapy, *PSA* prostate-specific antigen.

Percentages include participants with missing or invalid responses.

^a^Among participants consuming ≥4 drinks per week.

^b^Pre-tax annual household income from all sources in Australian dollars.

^c^Including Department of Veterans’ Affairs white or gold card.

^d^Body mass index ≥25 kgm^−2^.

^e^Weekly physical activity time <150 min, with each minute of walking or moderate physical activity counted as 1 min, and each minute of vigorous physical activity counted as 2 min, according to the Australian physical activity guidelines.^57^

^f^Excludes fruit juice.

^g^Serves of breakfast cereal and brown or wholemeal bread.

^h^All cancers combined analysis.

**Fig. 1:**
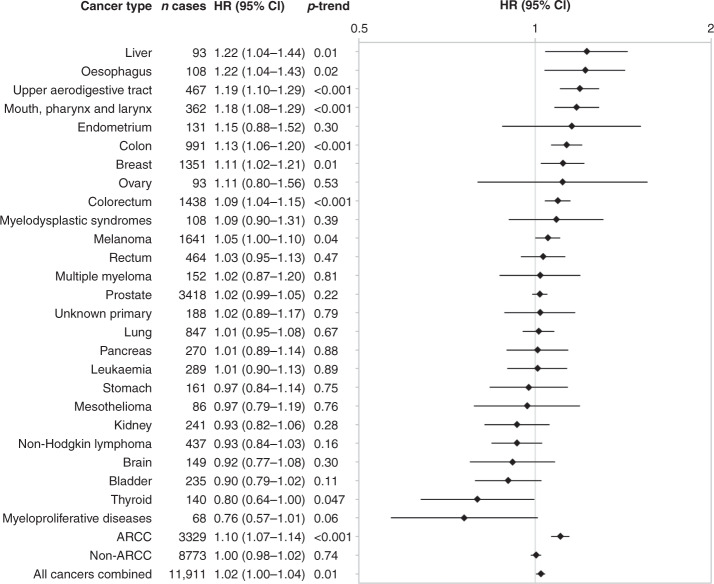
Hazard ratios (HR) and 95% confidence intervals (CI) of cancer risk per seven-drink increase in weekly alcohol consumption among drinkers in the 45 and Up Study (2006–2013). Linear trend calculated among drinkers only, where participants within each category of alcohol consumption at baseline were assigned the mean level of alcohol consumption they reported at first-wave follow-up (median 5.3 years after baseline). Models were adjusted for cancer-specific covariates as listed in Supplementary Table 1. Cancer cases do not sum to totals as some participants were diagnosed with two or more primary cancers. ARCC alcohol-related cancers combined.

Table 2 shows the risk of all cancer types in relation to weekly alcohol consumption. Compared to light drinkers, participants with higher levels of intake had significantly increased HRs for cancers of the upper aerodigestive tract, mouth, pharynx and larynx, oesophagus, colorectum, colon, liver, and female breast. The risk of all ARCC was significantly increased at >7 drinks per week. Among drinkers, there was also a significant positive trend of increased risk with increased levels of consumption for each of these cancer types, as well as a significant positive trend for melanoma and all cancers combined, and an inverse relationship with risk of thyroid cancer (Fig. 1). The risk of an IARC-determined, alcohol-related cancer increased by 10% with every additional seven drinks per week.

**Table 2:** Hazard ratios (HR) and 95% confidence intervals (CI) of cancer risk by alcohol consumption in the 45 and Up Study (2006–2013).

		HR drinks per week (95% CI)						
Cancer type (ICD-10 code)	Number of cases	0 to <1	≥1 to ≤3.5	>3.5 to ≤7	>7 to ≤14	>14 to ≤28	>28	*P*^a^
ARCC (C00–15; 18–20; 22; 32; 50^b^)	5111	0.97 (0.89–1.06)	1.00	0.97 (0.88–1.07)	1.11 (1.01–1.23)	1.17 (1.04–1.31)	1.41 (1.21–1.65)	<0.001
Upper aerodigestive tract (C00–15; 32)	652	0.92 (0.71–1.19)	1.00	0.80 (0.59–1.07)	1.01 (0.76–1.34)	1.18 (0.87–1.59)	1.88 (1.36–2.61)	<0.001
Mouth, pharynx and larynx (C00–14; 32)	486	0.77 (0.57–1.04)	1.00	0.73 (0.52–1.02)	1.07 (0.78–1.46)	1.19 (0.85–1.66)	1.80 (1.24–2.62)	<0.001
Oesophagus (C15)	171	1.55 (0.90–2.67)	1.00	1.07 (0.58–1.98)	0.90 (0.47–1.70)	1.21 (0.64–2.30)	2.11 (1.07–4.13)	0.01
Colorectum (C18–20)	2169	1.03 (0.90–1.18)	1.00	0.97 (0.83–1.14)	1.14 (0.98–1.33)	1.23 (1.03–1.46)	1.30 (1.03–1.62)	0.02
Colon (C18–19)	1521	1.02 (0.87–1.21)	1.00	0.99 (0.82–1.19)	1.09 (0.91–1.32)	1.29 (1.05–1.59)	1.45 (1.11–1.90)	0.01
Rectum (C20)	671	1.01 (0.78–1.29)	1.00	0.89 (0.67–1.18)	1.21 (0.92–1.58)	1.07 (0.79–1.44)	1.10 (0.75–1.62)	0.32
Liver (C22)	158	1.93 (1.08–3.47)	1.00	1.09 (0.54–2.17)	1.48 (0.76–2.86)	1.19 (0.57–2.50)	3.02 (1.49–6.13)	0.005
Breast (C50^b^)	2160	0.91 (0.80–1.03)	1.00	1.02 (0.89–1.17)	1.15 (1.00–1.34)	1.17 (0.97–1.42)^c^	–	0.003
Non-ARCC (all except C00–15; 18–20; 22; 32; 50^b^)	12,505	1.02 (0.96–1.08)	1.00	1.00 (0.94–1.07)	1.04 (0.98–1.11)	1.04 (0.97–1.11)	0.97 (0.89–1.07)	0.52
Stomach (C16)	247	1.15 (0.76–1.75)	1.00	1.16 (0.74–1.83)	1.18 (0.75–1.88)	1.01 (0.59–1.71)	0.88 (0.43–1.78)	0.91
Pancreas (C25)	419	1.09 (0.79–1.49)	1.00	1.09 (0.77–1.55)	1.31 (0.92–1.87)	1.23 (0.82–1.84)	0.92 (0.50–1.68)	0.60
Lung (C33–34)	1264	1.10 (0.91–1.33)	1.00	1.14 (0.93–1.41)	0.99 (0.80–1.22)	1.12 (0.89–1.40)	1.14 (0.86–1.50)	0.57
Melanoma (C43)	2272	0.91 (0.79–1.04)	1.00	0.95 (0.82–1.10)	1.09 (0.95–1.26)	1.08 (0.92–1.27)	1.08 (0.87–1.35)	0.051
Mesothelioma (C45)	132	1.47 (0.80–2.69)	1.00	1.05 (0.53–2.06)	1.80 (0.96–3.38)	0.92 (0.42–2.02)	0.94 (0.33–2.65)	0.15
Endometrium (C54.1)	285	1.46 (1.01–2.10)	1.00	0.98 (0.63–1.54)	1.21 (0.77–1.92)	1.19 (0.65–2.19)^c^	–	0.13
Ovary (C56)	163	0.74 (0.49–1.14)	1.00	0.61 (0.36–1.04)	0.87 (0.51–1.48)	1.14 (0.58–2.26)^c^	–	0.27
Prostate (C61)	4357	1.02 (0.92–1.14)	1.00	1.11 (1.00–1.24)	1.08 (0.97–1.20)	1.13 (1.01–1.27)	1.11 (0.96–1.27)	0.16
Kidney (C64)	354	1.11 (0.78–1.56)	1.00	1.24 (0.86–1.79)	1.07 (0.73–1.57)	0.86 (0.55–1.34)	0.85 (0.48–1.53)	0.51
Bladder (C67)	339	0.83 (0.60–1.16)	1.00	0.70 (0.48–1.01)	0.93 (0.65–1.33)	0.70 (0.46–1.06)	0.59 (0.32–1.08)	0.21
Brain (C71)	209	1.07 (0.68–1.69)	1.00	1.27 (0.80–2.03)	1.20 (0.75–1.94)	0.60 (0.32–1.11)	0.99 (0.49–2.04)	0.18
Thyroid (C73)	243	1.01 (0.71–1.44)	1.00	0.87 (0.58–1.32)	0.76 (0.49–1.19)	0.44 (0.22–0.87)	0.54 (0.19–1.52)	0.15
Non-Hodgkin lymphoma (C82–85)	665	0.98 (0.77–1.24)	1.00	0.83 (0.63–1.08)	0.98 (0.75–1.27)	0.81 (0.59–1.11)	0.71 (0.45–1.13)	0.35
Multiple myeloma (C90.0)	242	1.20 (0.80–1.80)	1.00	0.93 (0.59–1.47)	1.00 (0.63–1.59)	0.91 (0.53–1.56)	1.19 (0.60–2.36)	0.74
Leukaemia (C91–95)	428	1.00 (0.74–1.36)	1.00	1.10 (0.79–1.54)	0.98 (0.69–1.38)	0.97 (0.66–1.44)	1.05 (0.62–1.76)	0.97
Myeloproliferative diseases (D45; 47.1; 47.3–47.5)	106	0.98 (0.55–1.75)	1.00	0.97 (0.52–1.82)	0.72 (0.36–1.43)	0.75 (0.34–1.63)	0.17 (0.02–1.33)	0.53
Myelodysplastic syndromes (D46)	175	1.19 (0.73–1.94)	1.00	0.96 (0.56–1.66)	0.86 (0.48–1.53)	1.17 (0.64–2.14)	1.23 (0.54–2.81)	0.76
Unknown primary (C80)	298	0.86 (0.60–1.22)	1.00	0.76 (0.50–1.14)	0.89 (0.59–1.34)	0.81 (0.51–1.31)	1.06 (0.59–1.88)	0.77
All cancers combined (C00–97; D45–46; 47.1; 47.3–47.5)	17,332	1.00 (0.95–1.05)	1.00	0.99 (0.94–1.04)	1.05 (1.00–1.11)	1.06 (1.00–1.12)	1.04 (0.96–1.12)	0.054

*ICD-10* International Classification of Diseases, version 10, *ARCC* alcohol-related cancers combined.

Models were adjusted for cancer-specific covariates as listed in Supplementary Table 1. Cancer cases do not sum to totals as some participants were diagnosed with two or more primary cancers.

^a^*P* heterogeneity.

^b^Breast cancer in women only.

^c^> 14 drinks per week for women.

There were statistical interactions with sex (*P* = 0.04) and smoking status (*P* = 0.03) in relation to alcohol consumption for ARCC, but not for any individual cancer type. Risk was higher in women (HR: 1.12; 95% CI: 1.04–1.21) compared to men (1.08; 1.05–1.12), and in ever-smokers (1.16; 1.09–1.24) compared to never-smokers (1.06; 1.01–1.11).

**Fig. 2:**
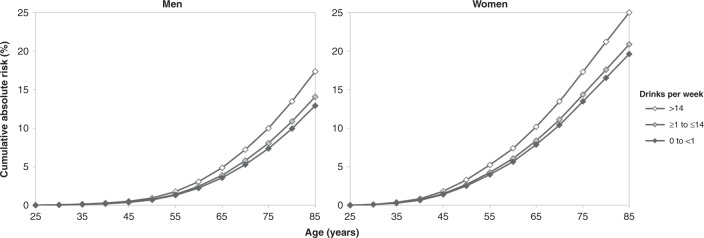
Estimated cumulative absolute risk (%) of an alcohol-related cancer diagnosis from age 25 to 85 years in Australia in 2014 by sex and level of alcohol consumption using hazard ratios from the 45 and Up Study (2006–2013). This calculation was based on the results of the continuous variable analysis among 45 and Up Study participants who consumed ≥1 drink per week. Three categories of drinking were used: 0 to <1 drink per week (never-drinkers, former drinkers and occasional drinkers who consumed <1 drink per week), ≥1 to ≤14 drinks per week (median 6 drinks in men and 5 drinks in women) and >14 drinks per week (median 21 drinks in men and 20 drinks in women). Note: Incidence data for alcohol-related cancers combined included International Classification of Diseases version 10 codes C30–31 while hazard ratio calculations did not.

Figure 2 shows the cumulative absolute risk of an alcohol-related cancer up to 85 years, by sex and level of alcohol consumption. By age 85 years, the absolute risk of an alcohol-related cancer was 17.3% in men and 25.0% in women for those consuming >14 drinks per week, compared to 12.9% in men and 19.6% in women for those consuming 0 to <1 drink per week. This represents an absolute risk increase of 4.4% in men and 5.4% in women. Detailed figures for each alcohol-related cancer type and for ARCC are provided in Supplementary Table 3.

**Fig. 3:**
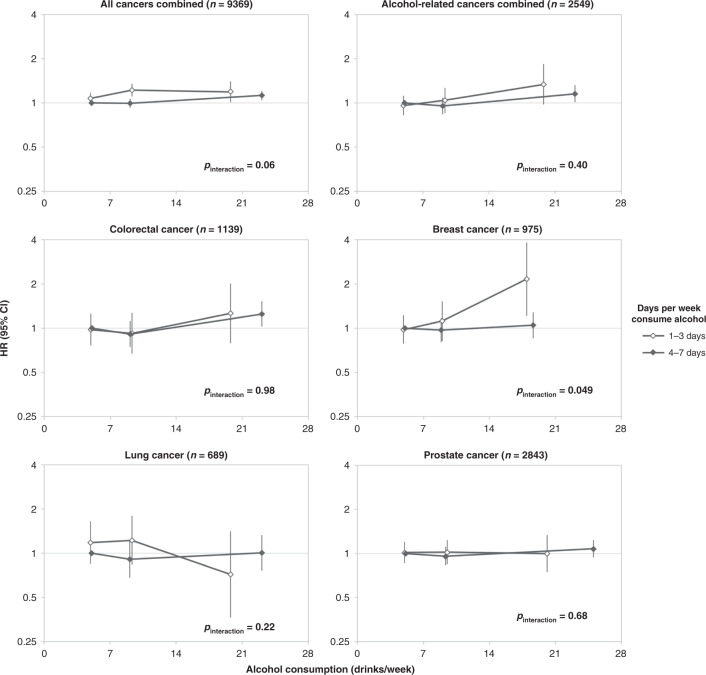
Hazard ratios (HR) and 95% confidence intervals (CI) of cancer risk by drinking pattern among participants consuming ≥ 4 drinks per week in the 45 and Up Study (2006–2013). Models were adjusted for cancer-specific covariates as listed in Supplementary Table 1. Reference category: ≥4 to <7 drinks per week, consumed on 4–7 days per week. Point estimates plotted at mean intake for each of the three levels of overall alcohol consumption (≥4 to <7 drinks per week, ≥7 to <14 drinks per week and ≥14 drinks per week). *P*_interaction_ is for test of interaction between days per week and drinks per week. Breast cancer in women only.

Risk of cancer in relation to drinking pattern is presented in Fig. 3. There was a marginally significant finding of a higher risk of breast cancer for women who consumed ≥14 drinks per week if those drinks were consumed on 1, 2 or 3 days per week rather than spread over 4–7 days per week. There was no evidence of interaction between days per week and drinks per week for other cancer types.

Overall, the results were not materially changed when the first year of follow-up was excluded, or when participants with self-reported melanoma were excluded. When the mean number of drinks reported at baseline rather than at follow-up was assessed in sensitivity analyses, HRs tended to be closer to the null, indicative of regression dilution. In sensitivity analyses using the actual number of drinks per week rather than the mean value of each category of intake, HRs tended to be closer to the null. The proportional hazard assumption was violated in some cases and the details of those results are addressed in the supplementary material.

## Discussion

In our study, the relative risk of incident cancers known to be caused by alcohol increased by 10% with every additional seven drinks per week, with consumption of more than 14 drinks a week estimated to account for a 4.4% higher absolute cumulative risk in men compared to consumption of 0 to <1 drink per week (i.e., 17.3% vs. 12.9%), and 5.4% higher risk in women (i.e., 25.0% vs. 19.6%), up to age 85 years. This finding is likely to be of significant public health importance, given that 16% of Australian adults were estimated to consume more than two standard drinks per day on average in 2017–2018.^1^ Consistent with the international evidence, cancers of the upper aerodigestive tract, mouth, pharynx and larynx, oesophagus, colorectum, colon, liver and breast, were each positively associated with overall alcohol consumption.^7,8,10,11,20^ Alcohol is also considered to be causally related to rectal cancer, and while this was not significantly associated with alcohol consumption in our study, the confidence intervals were compatible with prior evidence.^10^ Our study found mixed results for melanoma and thyroid cancer. A significant continuous positive association with alcohol intake was observed for melanoma among drinkers, but there were no significant differences between aggregated categories of intake when participants who consumed 0 to <1 drink per week were included. According to the WCRF, evidence for a relationship between alcohol consumption and melanoma risk in other studies is limited, with a meta-analysis suggesting a non-linear relationship, where risk increases up to ~10 g of alcohol per day and then plateaus.^10^ When analysed as a categorical variable including participants who consumed 0 to <1 drink per week, our results were consistent with this finding. Likewise, among drinkers, there was a significant continuous inverse trend in relation to thyroid cancer risk, but no significant variation when analysed as a categorical variable that included participants who consumed 0 to <1 drink per week. Meta-analyses have found a significant decreased risk of thyroid cancer for categories of intake, but no clear evidence of a linear dose–response relationship.^11,17^ Our results were also consistent with an inverse relationship for NHL and kidney cancer (although not statistically significant).^11,19^ When we examined the impact of drinking pattern on cancer risk, there was a marginally significant finding of an independent effect on the risk of breast cancer for any level of alcohol consumption that was consumed on few days of the week compared to an equivalent amount of intake consumed over many days of the week, but not for other cancer types.

Our results add to the evidence that alcohol consumption is an important risk factor for cancer in Australia. It was estimated that 2.8% of all cancers in Australia were attributed to alcohol consumption in 2010.^3^ Based on the relative risks reported here, the difference in the cumulative lifetime risk of diagnosis with an alcohol-related cancer between those consuming 0 to <1 drink per week and those consuming >14 drinks per week was found to be 4.4% and 5.4% for men and women, respectively. These estimates are higher than those reported for the British population;^58^ specifically, consumption of three bottles of wine per week (240 g of ethanol, or 24 Australian standard drinks) was associated with an increased absolute risk of cancer of 1.9% in men and 3.6% in women to age 80 years. This may reflect differences in relative risks used between the two studies, differences in population cancer incidence rates and that our study used an endpoint of 85 years rather than 80 years.

Previous studies have suggested that a heavy episodic ‘binge’ drinking pattern (defined in several different ways) may increase cancer risk, independent of the overall amount of alcohol consumed.^20,24,25,38^ Due to the questionnaire design, we were not able to examine heavy episodic drinking directly, but rather we examined whether participants who concentrated intake on 1–3 days per week had a different risk profile to those who consumed the same amount of alcohol on average over 4–7 days of the week. We report a marginally significant finding of an independent effect of pattern of drinking on breast cancer risk among women, suggestive of an increased risk in relation to ‘binge’ or ‘heavy episodic’ drinking (noting that our quantity-frequency construct can only approximate these behaviours, since we did not measure them directly). At least two previous studies have reported similar conclusions. One study of 74,854 American women (1988–2008) reported that the highest number of drinks consumed in one day in a typical month was associated with increased risk of breast cancer (*P*_trend_ = 0.04).^25^ Another study of 17,647 Danish women (1993–2001) reported that heavy episodic drinking (defined in several ways) increased breast cancer risk (although overall intake was not completely accounted for in that study).^30^ In contrast, null results in relation to drinking frequency have been reported in two studies.^25,34^ Overall, the evidence in relation to an association between heavy episodic drinking and breast cancer remains limited, and further research is required to understand the potential mechanistic basis of the relationship.

While we found potential evidence of an independent effect of pattern of drinking on risk for breast cancer, we note that our estimates for some outcomes were imprecise. For example, the confidence intervals for breast cancer and ARCC were compatible with substantial increased risk for those concentrating their intake on fewer days of the week. Although the association between drinking pattern and risk of all cancers combined was not statistically significant in this analysis (*P* = 0.06), it is possible that these relationships may change with longer follow-up and additional cases. The methods used in previous studies have differed greatly in the assessment of drinking patterns. Of studies that have accounted for overall alcohol consumption, it has been reported that consuming ≥5 drinks on drinking days was associated with increased pancreatic cancer risk,^24^ that the highest number of drinks consumed in one day in a typical month was associated with increased risk of ARCC in women,^20^ that higher intake per drinking day was associated with mortality from all cancers combined in men^38^ and that greater drinking frequency was associated with higher risk of ARCC in men^20^ and mortality from all cancers combined,^38^ while one study reported null results for ARCC^37^ in relation to drinking frequency. A number of studies that did not, or only partially, accounted for overall alcohol consumption, found that varying definitions of heavy episodic drinking increased risk for cancers of the stomach,^26^ lung^27^ and prostate^28,29^ and mortality from all cancers combined,^36^ while significant differences in the risk of mouth and pharyngeal cancer,^31^ mouth cancer,^32^ stomach cancer,^26^ prostate cancer^28^ and mortality from oesophageal cancer in men and pancreatic cancer in women^33^ have been reported in relation to drinking frequency, and some studies reported null results.^33,35^ As most studies of drinking pattern did not account for overall alcohol consumption, their findings are difficult to interpret, given that it is impossible to differentiate the effects of overall alcohol consumption from the impact of drinking pattern. If there is an effect of drinking pattern on cancer risk, it would be evidence of a mechanism where acute, heavy alcohol exposure impacts some body sites differently than chronic exposure, and that risk relationships may be missed if overall alcohol consumption is examined alone. Ultimately, well-designed and adequately powered studies that account for overall alcohol consumption are needed to address this research gap.

Our study has several strengths. It is a large prospective cohort study that used data linkage to obtain complete follow-up of health outcomes and allowed for systematic investigation of the impact of alcohol use on multiple cancer types simultaneously. In addition to overall alcohol consumption, we examined pattern of drinking, estimated absolute and relative risks and adjusted for a large number of potential confounders. We used age as the underlying time variable to minimise potential confounding by age. In addition, we used a reference group of very light drinkers as opposed to a reference group including non-drinkers, due to the potential for bias from the ‘sick-quitter effect’. This is important given recent Mendelian randomisation evidence which suggests that conventional epidemiological methods using non-drinkers as the reference group can result in U-shaped associations that are misinterpreted.^59^

One limitation of our study was that due to the questionnaire design, we were unable to distinguish the impact of never-drinking, former drinking and occasional drinking (<1 drink/week) on cancer risk, nor could we investigate differences by beverage type. Further, our drinking pattern analyses relied on a quantity-frequency construct rather than specific drinking patterns that were ascertained directly. Because the quantity-frequency construct was an average of the number of drinks consumed over the week, it could not be used to differentiate participants by daily changes in intake. For example, participants who consume a moderate amount of alcohol every day were grouped with those who drink heavily on the weekend and also consume small amounts during the week, even though the risk profile of these participants is likely to differ. Persons who participate in cohort studies may be more health conscious than the general population, and so heavier drinkers may be underrepresented in our study. Another limitation of our study was that we were not able to ascertain alcohol consumption and drinking patterns over the life course. For instance, there is evidence that alcohol consumption in young adulthood contributes to the accumulation of breast cancer risk in later life.^60^ Studies that measure overall alcohol consumption and drinking patterns from young adulthood may therefore find stronger relationships between alcohol consumption and breast cancer risk than we did in our study. It should also be noted that the proportion of participants who consumed alcohol at least weekly was higher in our study than in the 2007–2008 Australian National Health Survey for men but was similar for women. This may be due to the exclusion of participants who previously had cancer in our study, differences in sampling methods or differences in the specific wording of questions used to ascertain alcohol consumption.

A potential problem common to all observational studies measuring alcohol consumption is the tendency for participants to underreport intake.^61^ This could bias results, particularly if underreporting differs by consumption levels and drinking patterns. In both Australia and Canada, low-risk and non-heavy episodic drinkers were found to underreport consumption to a greater extent than higher-risk and heavy episodic drinkers.^62,63^ On the other hand, an English study found underreporting to be disproportionately associated with heavy drinking, frequent drinking and non-routine drinking compared to participants without these drinking behaviours.^64^ It is unclear how underreporting may impact our results; however, if indeed participants who were lighter drinkers underreported their alcohol consumption to a greater degree than heavier drinkers, HRs attributed to a given level of drinking may instead reflect higher levels of intake, with estimates for lighter drinkers being more vulnerable to this effect than those for heavier drinkers.

In Australia, interventions aimed at reducing the health risks of alcohol consumption have often focused on short-term harm minimisation. These include mass media campaigns, education programmes, industry-regulated responsible service of alcohol policies, ‘lockout’ laws and random breath testing. The focus of these measures has been on the short-term harms of heavy episodic drinking such as accidents and injury, and they are largely aimed at younger people. Our study demonstrates that the long-term harms of alcohol also affect older people in Australia, and it is important to target this population given evidence that more than half of risky drinkers aged ≥50 years in Australia do not perceive their level of drinking to be harmful, and identify as light, occasional or social drinkers.^65^ Evaluation of social marketing strategies has shown mixed results and challenges in influencing behaviour, particularly in younger people.^66^ However, a mass media campaign conducted in Australia in 2010 (*Alcohol and Cancer*), which focused on cancer rather than injury or other short-term harm, evaluated comparatively well among persons aged 18–64 years.^66^ Finally, interventions focusing on long-term harms such as cancer risk may be especially important during the COVID-19 pandemic, given recent reports that a portion of the Australian population has experienced an increase in alcohol consumption,^67,68^ with a risk that acquired drinking behaviour during COVID-19 may continue for a longer term.

The Australian alcohol consumption guidelines are about to change.^69^ Our results could help to inform future health guidelines by providing evidence that the relationship between drinking and cancer risk in Australia matches that of international evidence. Based on the results of our analysis, this may also include ongoing consideration of the evidence for pattern of drinking and breast cancer risk, particularly the avoidance of heavy episodic ‘binge’ drinking, which may be associated with increased risk above that related to the total amount consumed. Our findings also strengthen the case for adding cancer risk to alcoholic beverage labelling, targeted education programmes and for creating more informative national health guidelines for communication to the public about the prevention of chronic disease. Moreover, a general increase in awareness of the relationship between alcohol and cancer, at any age, may help to ‘de-normalise’ risky consumption across the life course. Our results strengthen the evidence associating alcohol consumption with cancer risk, and can be used in public health campaigns, to discourage risky alcohol consumption in Australia and internationally, with wider-ranging benefits to population health.

## Supplementary information

Supplementary information

## Data Availability

The data that support the findings of this study are available from the Sax Institute (https://www.saxinstitute.org.au/our-work/45-up-study/).
