# Supplementary material for: Alcohol consumption, drinking patterns and cancer incidence in an Australian cohort of 226,162 participants aged 45 years and over
Source: Br J Cancer. 2020 Oct 11;124(2):513–23. doi: 10.1038/s41416-020-01101-2 (PMC7853127; doi:10.1038/s41416-020-01101-2)
Supplement: Supplementary file 1 — Supplementary information [file 41416_2020_1101_MOESM1_ESM.docx]

**Supplementary information**

This supplementary file contains a table outlining the covariates included in each model, a table comparing alcohol consumption between participants included in the analysis and the Australian National Health Survey (2007-2008), a table of detailed cumulative absolute risk results, a description of the methods used to calculate cumulative absolute risk, and detailed information about the proportional hazards assumption violations.

**Supplementary Table 1. Covariates in models by cancer type in the 45 and Up Study (2006-2013).**

| **Cancer type (ICD-10 code)** | **Remoteness, education, household income, health insurance status, partner status, country of birth, smoking status and intensity, body mass index, physical activity** | **Sex** | **Fruit and vegetable intake** | **Fibre, red meat and processed meat intake** | **Time spent outdoors, skin tone** | **Parity and age at first birth, breastfeeding duration, menopausal status** | **HC use** | **MHT use** | **Aspirin use** | **Bowel, breast and prostate screening history** |
| --- | --- | --- | --- | --- | --- | --- | --- | --- | --- | --- |
| ARCC (C00-15;18-20;22;32;50^a^) | Yes | Yes | Yes | Yes | - | Yes | Yes | Yes | Yes | Bowel/Breast |
| Upper aerodigestive tract (C00-15;32) | Yes | Yes | Yes | - | - | - | - | - | Yes | - |
| - Mouth, pharynx and larynx (C00-14;32) | Yes | Yes | Yes | - | - | - | - | - | - | - |
| - Oesophagus (C15) | Yes | Yes | Yes | - | - | - | - | - | Yes | - |
| Colorectum (C18-20) | Yes | Yes | Yes | Yes | - | - | - | Yes | Yes | Bowel |
| Liver (C22) | Yes | Yes | - | - | - | - | Yes | - | - | - |
| Breast (C50^a^) | Yes | - | - | - | - | Yes | Yes | Yes | - | Breast |
| Non-ARCC (All except C00-15;18-20;22;32;50^a^) | Yes | Yes | Yes | - | Yes | Yes | Yes | Yes | - | Prostate |
| Stomach (C16) | Yes | Yes | Yes | - | - | - | - | - | - | - |
| Pancreas (C25) | Yes | Yes | - | - | - | - | - | - | - | - |
| Lung (C33-34) | Yes | Yes | Yes | - | - | - | - | - | - | - |
| Melanoma (C43) | Yes | Yes | - | - | Yes | - | - | - | - | - |
| Mesothelioma (C45) | Yes | Yes | - | - | - | - | - | - | - | - |
| Endometrium (C54.1) | Yes | - | - | - | - | Yes | Yes | Yes | - | - |
| Ovary (C56) | Yes | - | - | - | - | Yes | Yes | Yes | - | - |
| Prostate (C61) | Yes | - | - | - | - | - | - | - | - | Prostate |
| Kidney (C64) | Yes | Yes | - | - | - | - | - | - | - | - |
| Bladder (C67) | Yes | Yes | - | - | - | - | - | - | - | - |
| Brain (C71) | Yes | Yes | - | - | - | - | - | - | - | - |
| Thyroid (C73) | Yes | Yes | - | - | - | - | - | - | - | - |
| Non-Hodgkin lymphoma (C82-85) | Yes | Yes | - | - | - | - | - | - | - | - |
| Multiple myeloma (C90.0) | Yes | Yes | - | - | - | - | - | - | - | - |
| Leukaemia (C91-95) | Yes | Yes | - | - | - | - | - | - | - | - |
| Myeloproliferative diseases (D45;47.1;47.3-47.5) | Yes | Yes | - | - | - | - | - | - | - | - |
| Myelodysplastic syndromes (D46) | Yes | Yes | - | - | - | - | - | - | - | - |
| Unknown primary (C80) | Yes | Yes | - | - | - | - | - | - | - | - |
| All cancers combined (C00-97;D45-46;47.1;47.3-47.5) | Yes | Yes | Yes | Yes | Yes | Yes | Yes | Yes | Yes | All three |

All models adjusted for age through use of age as the underlying time variable. Remoteness categories: Major city; Inner regional; Outer regional; Remote. Highest level of education categories: No school certificate or other qualifications; school or intermediate certificate; Higher school or leaving certificate; Trade/apprenticeship; Certificate/diploma; University degree or higher. Annual household income in Australian dollars categories: <$30 000; ≥$30 000 to <$70 000; ≥$70 000. Health insurance status categories: Private health insurance – with extras; Private health insurance – without extras; Department of Veterans’ Affairs white or gold card; Health care concession card; None of these. Partner status categories: Married/living with partner; Not married/living with partner. Country of birth categories: Australia; Canada/Ireland/ New Zealand/United Kingdom/United States of America; Other country. Smoking status and intensity categories: Never-smoker; Ex-smoker (≤15 cigarettes/day); Ex-smoker (>15 cigarettes/day); Ex-smoker (missing cigarettes/day); Current smoker (≤15 cigarettes/day); Current smoker (>15 cigarettes/day); Current smoker (missing cigarettes/day). Body mass index categories: Underweight (BMI <18.5 kgm^-2^); Normal range (BMI ≥18.5 to <25 kgm^-2^); Overweight (BMI ≥25 to <30 kgm^-2^); Obese (BMI ≥30 kgm^-2^). Physical activity categories: Inactive (0 minutes/week); Insufficient (>0 to <150 minutes/week); Sufficient (≥150 to <300 minutes/week); High (≥300 minutes/week); with each minute of walking or moderate physical activity counted as 1 minute, and each minute of vigorous physical activity counted as 2 minutes, according to the Australian physical activity guidelines^1^. Sex categories: Male; Female. Fruit intake categories: <1 serve/day; ≥1 to <2 serves/day; ≥2 serves/day. Vegetable intake categories: <3 serves/day; ≥3 to <5 serves/day; ≥5 serves/day. Fibre intake categories: <7 serves/week; ≥7 to <14 serves/week; ≥14 to <21 serves/week; ≥21 serves/week. Red meat intake categories: 0 times/week; >0 to ≤2 times/week; >2 to ≤5 times/week; >5 times/week. Processed meat categories: 0 times/week; >0 to ≤1 times/week; >1 to ≤2 times/week; >2 times/week. Time spent outdoors categories: <2 hours/day; ≥2 to <4 hours/day; ≥4 to <6 hours/day; ≥6 hours/day. Skin tone categories: Fair; Olive; Brown or black. Parity and age at first birth categories: No children; 1 child (<25 years); 1 child (≥25 years); 2 children (<25 years); 2 children (≥25 years); ≥3 children (<25 years); ≥3 children (≥25 years); Male indicator. Breastfeeding duration categories: Never breastfed; >0 to ≤12 months; >12 to ≤24 months; >24 months; Male indicator. Menopausal status categories: Pre-menopausal; Irregular periods; Post-menopausal; Male indicator. Hormonal contraceptive use categories: Never used; Ever used; Male indicator. Menopausal hormone therapy use categories: Never used; Formerly used; Current user; Male indicator. Aspirin use categories: No; Yes. Bowel screening history categories: Not in the last 10 years; Yes, ≥2 to ≤10 years ago; Yes, <2 years ago. Breast screening history categories: Not in the last 10 years; Yes, ≥2 to ≤10 years ago; Yes, <2 years ago; Male indicator. Prostate screening history categories: Never; Yes, 1-3 times; Yes, >3 times; Yes, times missing; Female indicator. All categorical covariates except sex had a missing indicator category. ^a^Breast cancer in women only. ICD-10, International Classification of Diseases, version 10. HC, Hormonal Contraceptive. MHT, Menopausal Hormone Therapy. ARCC, Alcohol-Related Cancers Combined. BMI, Body Mass Index.

**Supplementary Table 2. Comparison of alcohol consumption by sex and age between participants included in the analysis from the 45 and Up Study baseline survey (2006-2009) and the Australian National Health Survey (2007-2008).**

| **Sex and age** | **Alcohol consumption at least weekly (%)** | |
| --- | --- | --- |
|  | **Participants included in the analysis**  **from the baseline survey of the 45**  **and Up Study (2006-2009)** | **Australian National**  **Health Survey**  **(2007-2008)^a^** |
| **Men** |  |  |
| 45-54 years | 78.8 | 76.0 |
| 55-64 years | 79.6 | 72.5 |
| 65-74 years | 75.8 | 68.2 |
| ≥75 years | 68.8 | 60.2 |
| **Women** |  |  |
| 45-54 years | 64.5 | 61.9 |
| 55-64 years | 61.5 | 60.6 |
| 65-74 years | 53.8 | 52.8 |
| ≥75 years | 43.7 | 45.4 |

Proportions exclude missing data. ^a^Results weighted by age, sex, area of usual residence and probability of selection in household to match Australian Bureau of Statistics estimates for December 2007, and excluded persons living in very remote areas and residents of hotels, motels, caravan parks and institutions.

**Supplementary Table 3. Cumulative absolute risk (%) of cancer diagnosis from age 25 to 85 years in Australia in 2014 by sex and level of alcohol consumption using hazard ratios from the 45 and Up Study (2006-2013).**

| **Sex and age** | **Mouth, pharynx and larynx cancer^a^ (drinks/week)** | | |  | **Oesophageal cancer**  **(drinks/week)** | | | |  | | **Colorectal cancer**  **(drinks/week)** | | |  | **Liver cancer**  **(drinks/week)** | | |  | **Breast cancer**  **(drinks/week)** | | |  | **ARCC^a^**  **(drinks/week)** | | |
| --- | --- | --- | --- | --- | --- | --- | --- | --- | --- | --- | --- | --- | --- | --- | --- | --- | --- | --- | --- | --- | --- | --- | --- | --- | --- |
|  | **0 to <1** | **≥1 to ≤14** | **>14** |  | **0 to <1** | **≥1 to ≤14** | **>14** |  | | **0 to <1** | | **≥1 to ≤14** | **>14** |  | **0 to <1** | **≥1 to ≤14** | **>14** |  | **0 to <1** | **≥1 to ≤14** | **>14** |  | **0 to <1** | **≥1 to ≤14** | **>14** |
| **Men** |  |  |  |  |  |  |  |  | |  | |  |  |  |  |  |  |  |  |  |  |  |  |  |  |
| 25 years | 0.00 | 0.00 | 0.00 |  | 0.00 | 0.00 | 0.00 |  | | 0.00 | | 0.00 | 0.00 |  | 0.00 | 0.00 | 0.00 |  | - | - | - |  | 0.00 | 0.00 | 0.00 |
| 30 years | 0.01 | 0.01 | 0.01 |  | 0.00 | 0.00 | 0.00 |  | | 0.03 | | 0.03 | 0.03 |  | 0.00 | 0.00 | 0.00 |  | - | - | - |  | 0.03 | 0.04 | 0.05 |
| 35 years | 0.03 | 0.03 | 0.05 |  | 0.00 | 0.00 | 0.00 |  | | 0.07 | | 0.07 | 0.09 |  | 0.00 | 0.00 | 0.00 |  | - | - | - |  | 0.10 | 0.11 | 0.14 |
| 40 years | 0.05 | 0.06 | 0.08 |  | 0.00 | 0.00 | 0.00 |  | | 0.12 | | 0.13 | 0.16 |  | 0.01 | 0.01 | 0.02 |  | - | - | - |  | 0.18 | 0.20 | 0.25 |
| 45 years | 0.11 | 0.13 | 0.19 |  | 0.01 | 0.01 | 0.01 |  | | 0.22 | | 0.23 | 0.28 |  | 0.02 | 0.03 | 0.04 |  | - | - | - |  | 0.37 | 0.40 | 0.50 |
| 50 years | 0.21 | 0.24 | 0.34 |  | 0.02 | 0.03 | 0.04 |  | | 0.37 | | 0.40 | 0.48 |  | 0.05 | 0.05 | 0.08 |  | - | - | - |  | 0.67 | 0.73 | 0.92 |
| 55 years | 0.39 | 0.45 | 0.64 |  | 0.05 | 0.06 | 0.10 |  | | 0.65 | | 0.70 | 0.84 |  | 0.12 | 0.14 | 0.22 |  | - | - | - |  | 1.27 | 1.39 | 1.75 |
| 60 years | 0.67 | 0.76 | 1.08 |  | 0.10 | 0.12 | 0.19 |  | | 1.06 | | 1.14 | 1.38 |  | 0.26 | 0.31 | 0.48 |  | - | - | - |  | 2.23 | 2.44 | 3.06 |
| 65 years | 0.98 | 1.12 | 1.59 |  | 0.18 | 0.22 | 0.33 |  | | 1.77 | | 1.91 | 2.31 |  | 0.41 | 0.49 | 0.76 |  | - | - | - |  | 3.55 | 3.89 | 4.87 |
| 70 years | 1.35 | 1.55 | 2.19 |  | 0.31 | 0.37 | 0.56 |  | | 2.78 | | 3.00 | 3.61 |  | 0.56 | 0.66 | 1.02 |  | - | - | - |  | 5.27 | 5.76 | 7.19 |
| 75 years | 1.72 | 1.97 | 2.79 |  | 0.47 | 0.56 | 0.85 |  | | 4.09 | | 4.41 | 5.31 |  | 0.75 | 0.90 | 1.38 |  | - | - | - |  | 7.36 | 8.04 | 10.00 |
| 80 years | 2.13 | 2.44 | 3.45 |  | 0.68 | 0.81 | 1.22 |  | | 5.85 | | 6.30 | 7.57 |  | 0.97 | 1.16 | 1.78 |  | - | - | - |  | 9.96 | 10.86 | 13.46 |
| 85 years | 2.62 | 3.00 | 4.23 |  | 0.90 | 1.07 | 1.62 |  | | 7.90 | | 8.49 | 10.18 |  | 1.23 | 1.46 | 2.24 |  | - | - | - |  | 12.90 | 14.05 | 17.35 |
| **Women** |  |  |  |  |  |  |  |  | |  | |  |  |  |  |  |  |  |  |  |  |  |  |  |  |
| 25 years | 0.00 | 0.00 | 0.00 |  | 0.00 | 0.00 | 0.00 |  | | 0.00 | | 0.00 | 0.00 |  | 0.00 | 0.00 | 0.00 |  | 0.00 | 0.00 | 0.00 |  | 0.00 | 0.00 | 0.00 |
| 30 years | 0.01 | 0.01 | 0.01 |  | 0.00 | 0.00 | 0.00 |  | | 0.03 | | 0.03 | 0.04 |  | 0.00 | 0.00 | 0.00 |  | 0.04 | 0.04 | 0.05 |  | 0.07 | 0.08 | 0.10 |
| 35 years | 0.02 | 0.02 | 0.03 |  | 0.00 | 0.00 | 0.00 |  | | 0.07 | | 0.08 | 0.09 |  | 0.00 | 0.00 | 0.00 |  | 0.17 | 0.18 | 0.23 |  | 0.26 | 0.28 | 0.35 |
| 40 years | 0.04 | 0.04 | 0.06 |  | 0.00 | 0.00 | 0.00 |  | | 0.12 | | 0.13 | 0.16 |  | 0.01 | 0.01 | 0.01 |  | 0.46 | 0.49 | 0.62 |  | 0.62 | 0.67 | 0.82 |
| 45 years | 0.06 | 0.07 | 0.10 |  | 0.00 | 0.00 | 0.00 |  | | 0.20 | | 0.22 | 0.26 |  | 0.01 | 0.01 | 0.02 |  | 1.08 | 1.16 | 1.47 |  | 1.36 | 1.46 | 1.79 |
| 50 years | 0.10 | 0.11 | 0.15 |  | 0.00 | 0.00 | 0.00 |  | | 0.34 | | 0.37 | 0.44 |  | 0.02 | 0.02 | 0.03 |  | 2.02 | 2.18 | 2.74 |  | 2.49 | 2.67 | 3.27 |
| 55 years | 0.16 | 0.18 | 0.25 |  | 0.01 | 0.01 | 0.02 |  | | 0.58 | | 0.62 | 0.74 |  | 0.04 | 0.04 | 0.07 |  | 3.20 | 3.45 | 4.33 |  | 3.99 | 4.26 | 5.21 |
| 60 years | 0.24 | 0.27 | 0.38 |  | 0.03 | 0.04 | 0.06 |  | | 0.87 | | 0.93 | 1.12 |  | 0.07 | 0.08 | 0.12 |  | 4.45 | 4.80 | 6.01 |  | 5.65 | 6.04 | 7.37 |
| 65 years | 0.34 | 0.38 | 0.53 |  | 0.06 | 0.07 | 0.10 |  | | 1.39 | | 1.48 | 1.78 |  | 0.12 | 0.13 | 0.21 |  | 5.99 | 6.45 | 8.07 |  | 7.84 | 8.37 | 10.19 |
| 70 years | 0.46 | 0.52 | 0.74 |  | 0.11 | 0.13 | 0.20 |  | | 2.03 | | 2.16 | 2.61 |  | 0.16 | 0.19 | 0.29 |  | 7.77 | 8.37 | 10.43 |  | 10.40 | 11.10 | 13.47 |
| 75 years | 0.61 | 0.68 | 0.96 |  | 0.18 | 0.20 | 0.31 |  | | 2.99 | | 3.18 | 3.84 |  | 0.24 | 0.27 | 0.42 |  | 9.73 | 10.46 | 13.01 |  | 13.44 | 14.33 | 17.31 |
| 80 years | 0.77 | 0.87 | 1.23 |  | 0.26 | 0.30 | 0.46 |  | | 4.43 | | 4.72 | 5.68 |  | 0.35 | 0.40 | 0.62 |  | 11.29 | 12.14 | 15.05 |  | 16.53 | 17.60 | 21.17 |
| 85 years | 0.93 | 1.05 | 1.48 |  | 0.36 | 0.42 | 0.64 |  | | 6.03 | | 6.42 | 7.71 |  | 0.49 | 0.56 | 0.86 |  | 12.75 | 13.70 | 16.95 |  | 19.63 | 20.87 | 24.99 |

This calculation was based on the results of the continuous variable analysis among 45 and Up Study participants consuming ≥1 drink per week. Three categories of drinking were used: 0 to <1 drink per week (never-drinkers, former drinkers and occasional drinkers who consumed <1 drink per week), ≥1 to ≤14 drinks per week (median 6 drinks in men and 5 drinks in women), and >14 drinks per week (median 21 drinks in men and 20 drinks in women. The sums of cumulative risks across individual cancer types may differ from the corresponding risk for ARCC because: i) Some Australians were diagnosed with two or more different alcohol-related cancers while the risk of ARCC in the 45 and Up Study represents the risk of first diagnosis of alcohol-related cancer only; ii) The HR used for ARCC was sex-specific due to the detection of an interaction between alcohol consumption and sex for ARCC risk, while the HRs used for individual cancer types were not sex-specific. ^a^Incidence data included International Classification of Diseases version 10 codes C30-31 while hazard ratio calculations did not. ARCC, Alcohol-Related Cancers Combined.

**Cumulative absolute risk**

The hazard ratios obtained from the continuous variable analysis, the 2017-18 National Health Survey sex- and age-specific alcohol consumption prevalence data^2^ and sex- and age-specific 2014 national cancer incidence data^3^ were used to calculate the cumulative absolute risk of cancer in Australians from age 25 to 85 years in 2014 by alcohol consumption status. These datasets were the most recent years available. This method was used in a previous study examining smoking status and cumulative risk of mortality in the 45 and Up Study^4^. Cumulative risk was calculated by sex for three drinking groups: Persons consuming 0 to <1 drink per week (never-drinkers, former drinkers and occasional drinkers who consumed <1 drink per week), persons consuming >1 to ≤14 drinks per week, and persons consuming >14 drinks per week. Age groups in five-year increments were used: 25-29 years, 30-34 years … 80-84 years. In each sex and age group, the absolute rates of a specific cancer in persons consuming >14 drinks per week (A_>14_), persons consuming 0 to <1 drink per week (A_0_) and persons consuming ≤ 14 drinks per week (A_≤14_) were given by:

A_>14_ = A / (P_>14_ + P_≤14_ x HR_≤14_ / HR_>14_ + P_0_ / HR_>14_)

A_0_ = A / (P_>14_ x HR_>14_ + P_≤14_ x HR_≤14_ + P_0_)

A_≤14_ = A_0_ x HR_≤14_

Where A = the Australian incidence rate for this cancer type in this sex and age group, P_0_ = the national prevalence of persons consuming 0 to <1 drink per week for this sex and age group, P_≤14_ = the national prevalence of persons consuming >1 to ≤14 drinks per week for this sex and age group, P_>14_ = the national prevalence of persons consuming >14 drinks per week for this sex and age group, HR_≤14_ = the hazard ratio for this cancer type in persons consuming >1 to ≤14 drinks per week compared to persons consuming 0 to <1 drink per week derived from the continuous variable analysis of weekly alcohol consumption (calculated for the median drinker within this group in the 45 and Up Study: 6 drinks per week for men and 5 drinks per week for women), and HR_>14_ = the hazard ratio for this cancer type in persons consuming >14 drinks per week compared to persons consuming 0 to <1 drink per week derived from the continuous variable analysis of weekly alcohol consumption (calculated for the median drinker within this group in the 45 and Up Study: 21 drinks per week for men and 20 drinks per week for women). For alcohol-related cancers combined sex-specific HRs were used, as an interaction between alcohol consumption and sex was detected in the main analysis for this outcome. Non-sex-specific HRs were used for each individual cancer type. Absolute incidence for each cancer type was then calculated for each sex and five-year age group ($i$), by:

1 - exp(-5 $\sum_{i=(25-29)}^{x} A_{i}$)

Where *x* = age 30, 35 … 85 years, and A_i_ = A_0_, A_≤14_ or A_>14_ for each five-year age group. These were then summed to calculate the sex-specific cumulative absolute risk of cancer from age to 25 and *x* by drinking status. This was performed for cancers of the mouth, pharynx and larynx, oesophagus, colorectum, liver, female breast and alcohol-related cancers combined. The results were graphed for colorectal, breast and alcohol-related cancers combined.

It should be noted that the continuous variable analysis excluded non-drinkers, and that the calculation of cumulative absolute risk in non-drinkers assumed that the increased cancer risk per seven drink increase in weekly alcohol consumption can be extrapolated to non-drinkers. An alternative approach would be to include non-drinkers in the continuous variable analysis, however this would potentially increase the level of bias in the absolute risk estimates due to the problem of the ‘sick-quitter effect’ among non-drinkers. This is a vexed problem, and we have decided to exclude non-drinkers from the continuous variable analysis when calculating cumulative absolute risk as we consider these values likely to be less biased.

Some other limitations should also be noted. Firstly, this analysis assumes that the relationship between alcohol consumption and cancer risk does not differ by age, enabling cancer risk attributable to drinking between the ages of 25 and 45 years to be estimated. Secondly, the level of alcohol consumption of the median participant consuming >14 drinks per week in the 45 and Up Study may be lower than that of the Australian population if the 45 and Up Study is a ‘healthy cohort’. This would result in an underestimation of the level of risk for the >14 drinks per week group. To a lesser extent differences in the median level of alcohol consumption between participants in the 45 and Up Study and the Australian population may also bias estimates for the >1 to ≤14 drinks per week group. Finally, due to the available cancer incidence data, incidence data for mouth, pharynx and larynx cancer and alcohol related cancers combined included International Classification of Diseases version 10 codes C30-31 while the hazard ratio calculations did not.

**Proportional hazards assumption violations**

Statistically significant violations of the proportional hazards assumption were detected for colon cancer (*p* = 0.003; remoteness, education, partner status, smoking status and intensity, fibre intake, MHT use), endometrial cancer (*p* = 0.02; alcohol consumption, education, country of birth, smoking status and intensity, body mass index, breastfeeding duration, MHT use), prostate cancer (*p* = 0.03; education, partner status, prostate screening history), melanoma (*p* <0.001; sex, remoteness, education, health insurance status, country of birth), alcohol-related cancers combined (*p* <0.001; sex, education, country of birth, smoking status and intensity, fibre intake, MHT use), non-alcohol-related cancers combined (*p* = 0.002; sex, partner status, smoking status and intensity, skin tone, parity and age at first birth, prostate screening history), and all cancers combined (*p* <0.001; sex, education, partner status, smoking status and intensity, parity and age at first birth, menopausal status, MHT use, prostate screening history).

For most variables with a statistically significant proportional hazards assumption violation plotting the log-log graphs did not reveal obvious proportional hazards violations (results not shown). The exceptions to this were remoteness for colon cancer, alcohol consumption, education, smoking status and intensity and MHT use for endometrial cancer, and smoking status and intensity and fibre intake for alcohol-related cancers combined. In each of these cases, the results were not materially altered in the stratified Cox models (results not shown). There was perhaps some suggestion that for endometrial cancer, the relative risks of moderate to heavy drinking were higher at older ages compared to younger ages; however the overall alcohol relative risks for age strata of <60 and >=60 years were not significant on their own. Therefore, models without stratification were reported for ease of interpretation.

**References**

1. Australian Government Department of Health. Make your Move - Sit less – Be active for life! (Australian Government Department of Health, Canberra, 2014).

2. Australian Bureau of Statistics. *National Health Survey: First Results 2017–18* (ABS, Canberra, 2018).

3. Australian Institute of Health and Welfare. *Cancer data in Australia, ACIM Books*. <https://www.aihw.gov.au/reports/cancer/cancer-data-in-australia/acim-books> (2018).

4. Banks, E., Joshy, G., Weber, M. F., Liu, B., Grenfell, R., Egger, S. et al. Tobacco smoking and all-cause mortality in a large Australian cohort study: findings from a mature epidemic with current low smoking prevalence. *BMC Med*. **13**, 38 (2015).
